# Evaluating a Program Addressing the Certified Nursing Assistant (CNA) and Home Health Aide (HHA) Workforce Shortage

**DOI:** 10.1093/geroni/igaf122.2337

**Published:** 2025-12-31

**Authors:** Brittany McFeeley, Natasha Bryant, Verena Cimarolli, Robyn Stone

**Affiliations:** University of Massachusetts Boston, Boston, Massachusetts, United States; LeadingAge, Washington, District of Columbia, United States; LeadingAge, Washington, District of Columbia, United States; LeadingAge, Washington, District of Columbia, United States

## Abstract

The Gateway-In Project© (TGIP©) was established to expand the CNA and HHA workforce by recruiting students, providing free training, supports, and job placement assistance for new CNAs and HHAs in California. To assess TGIP©’s impact, we surveyed Year 1 graduates for employment status and career plans 12-18 months post-graduation. We also surveyed Year 2 students before (baseline) and after training completion to determine changes in students’ views of working in aging services, their financial and employment status. Students of both cohorts were mostly women of color, 35 years or younger, and had low household incomes. Over 80% of Year 1 students (N = 271), were employed as CNAs, HHAs, or both with close to 70% working in skilled nursing facilities. Working as a CNA/HHA was predominantly (74%) perceived as “a steppingstone to furthering their career” over “a career” or “job to make money.” Most students (73%) reported planning to advance to another occupation, particularly to a higher nursing degree/occupation. Year 2 students (N = 146) showed significantly lower interest in working as a CNA/HHA (t=-4.923, p=.004) and in working with older adults (t=-2.78, p=.006) after training completion compared to baseline. There was a significant increase in household income (t = 2.140, p=.034). Most were employed as CNAs. TGIP© evaluation demonstrates employment gains, important career advancement aspirations, and increased household incomes. Decreases in interest in working as CNAs/HHAs and with older adults may be due to the job’s demands becoming clearer during training. The ongoing evaluation efforts will provide further insights into TGIP©’s long-term impact.

